# Electron Microscopy Provides Quantitative Insights into Modified Magnetic Bead Layers for Electrochemical Immunoassays

**DOI:** 10.1021/acs.bioconjchem.5c00616

**Published:** 2026-04-21

**Authors:** Hannah A. Richards, William R. Lowery, Andrea K. Locke, David E. Cliffel

**Affiliations:** † Department of Chemistry, Vanderbilt University, Nashville, Tennessee 37235, United States; ‡ Department of Chemistry and Department of Biomedical Engineering, Vanderbilt University, Nashville, Tennessee 37235, United States

## Abstract

Sensitive cytokine quantification is essential for understanding inflammatory disease mechanisms and guiding diagnostic development. Here, we characterize a magnetic bead electrochemical sandwich assay (MBESA) based upon 2.8 μm magnetic Dynabeads for the rapid electrochemical detection of the inflammatory cytokine interleukin-1β (IL-1β), a key mediator of immune signaling. Scanning transmission electron microscopy coupled with energy-dispersive X-ray spectroscopy (STEM-EDX) provided high-resolution visualization and quantitative mapping of conjugated MBESA layers. Incorporation of streptavidin-gold nanospheres provided direct, nanoscale evidence of antibody and antigen bioconjugation, revealing binding heterogeneity marked by a triplicate increase in sulfur content. This work demonstrates precise quantification of assay bioconjugation, addressing a critical gap in the structural characterization of bead-based immunoassays. By bridging biomolecule arrangement to assay performance, the MBESA positions itself as a leading electrochemical platform for inflammatory cytokine detection.

## Graphical abstract

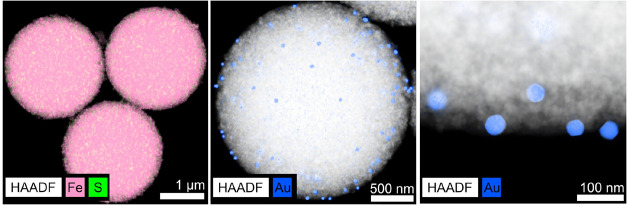

## Introduction

Modern innovations have guided researchers toward the development of unique protein bioconjugations for new disease diagnostic techniques such as electrochemical immunoassay sensors.^1,2^ Electrochemical immunoassay sensors often incorporate the use of antibodies, antigens, enzymes, and nanomaterials coupled with electrochemical techniques to precisely quantify the concentration of target analytes.^3^ Immunosensors use antibodies as biorecognition elements to translate the antibody–antigen binding event into a measurable signal.^4^ Electrochemical methods to obtain a readout signal, including amperometry, cyclic voltammetry (CV), square wave voltammetry (SWV), and electrochemical impedance spectroscopy (EIS), are increasingly common for bioconjugation applications for their ease-of-use, affordability, and rapid results.^5^ With an increase in the development of bioconjugated immunoassays and electrochemical immunosensors, it is highly important to investigate the various components of the bioconjugation process to maximize sensor optimization for the fabrication of site-specific electrochemical biosensors.

Magnetic beads have emerged as highly promising platforms for immunoassays, with applications spanning enzyme-linked immunosorbent assays (ELISAs), point-of-care (POC) diagnostics, and advanced biosensing formats. Recent developments demonstrate their broad utility in microfluidic systems,^6^ complex protein detection,^7^ influenza A detection,^8^ Raman reporter labels,^9^ CRISPR/Cas12a sandwich assays,^10^ nanobody modifications,^11^ and electrochemical sensors.^12^ Their versatility, simple magnetic separation, high surface-to-volume ratio, and compatibility with small sample volumes make magnetic bead-based immunoassays increasingly attractive.^13^ Despite their adoption and the crucial role they play in various diagnostic applications, a critical gap remains in the comprehensive, microscopic characterization of these intricate systems. While select reports have included surface and substrate characterization,^8^ detailed high-resolution microscopy investigations, particularly using techniques like scanning electron microscopy (SEM) and transmission electron microscopy (TEM), are rare in the literature and have yet to be applied to electrochemical magnetic bead immunoassays. This lack of structural insight limits our understanding of bead architecture, surface modification processes, and multilayer assembly, all of which are crucial for optimizing sensor performance and identifying mechanistic limitations.

Magnetic Dynabeads (2.8 μm diameter, Thermo Fisher Scientific) represent one of the most widely adopted bead platforms for such applications. These monodisperse, hydrophilic beads consist of a superparamagnetic iron oxide core encased within a polystyrene coating functionalized with surface activated epoxy groups.^14^ They are fabricated through an emulsion based polymerization process in which magnetite nanoparticles are dispersed and encapsulated within a growing polystyrene matrix, forming highly uniform superparamagnetic microspheres prior to epoxy functionalization.^14^ Their epoxy chemistry enables covalent attachment of proteins through nucleophilic attack by primary amines via an S_N_2 reaction mechanism, facilitating stable biomolecule immobilization. The unique architecture of these beads provides a functional surface area that exceeds what would be expected from their geometric diameter alone. Although the particles appear smooth at the microscale, the polymer-epoxy coating presents a highly accessible three-dimensional reactive landscape, allowing a greater density of biomolecules to immobilize than on a flat spherical surface. Thermo Fisher Scientific further highlights that Dynabeads promote favorable antibody orientation, contributing to improved assay sensitivity and reproducibility. Although their biochemical coupling chemistry is well described, detailed structural interrogation of Dynabeads following immunoassay layer assembly remains limited, reinforcing the need for systematic high-resolution microscopy studies.^15^

Our laboratory has recently investigated the use of magnetic bead-based electrochemical immunoassay sensors for interleukin-6 (IL-6)^12^ and interleukin-1β (IL-1β) detection in organ on-a-chip (OoC) applications.^16^ Despite extensive study in many organ systems, key aspects of cytokine signaling and physiology remain unexplored.^17^ In particular, there is a growing demand to investigate inflammatory cytokines that act as critical mediators in disease progression and tissue dysfunction, including in gestational membranes.^18^ IL-1β is a key mediator of immune signaling that exhibits significant concentration changes in pathological contexts.^19^ Our recently developed magnetic bead electrochemical sandwich assay (MBESA)^16^ was constructed based upon antibody–antigen bioconjugation to quantify IL-1β, and additional inflammatory cytokines, for robust applications. Immunoassays such as the MBESA provide a powerful alternative to conventional methods for cytokine detection, offering the potential for earlier and more precise diagnostic evaluation.

The MBESA is an electrochemical immunoassay based upon amperometric quantification designed to rapidly detect target analyte in complex biological samples. In this investigation, the MBESA design consists of intricate layers of magnetic Dynabeads, IL-1β capture antibody, bound IL-1β antigen, IL-1β detection antibody, horseradish-peroxidase (HRP), and 3,3′,5,5′-tetramethylbenzidine (TMB), shown in Figure 1, to generate an electrochemical signal and quantification of IL-1β. While the fundamental design and analytical performance of the MBESA for IL-1β has been previously established by our laboratory,^16^ the present work distinguishes itself by focusing on the in-depth microscopic characterization of the magnetic beads and conjugated immunoassay. This work addresses this critical need for high-resolution imaging to confirm and quantify the conjugation chemistry on the surface of the magnetic beads, providing unprecedented visual and quantitative data that was not the primary focus of our previous publications.

**Figure 1:**
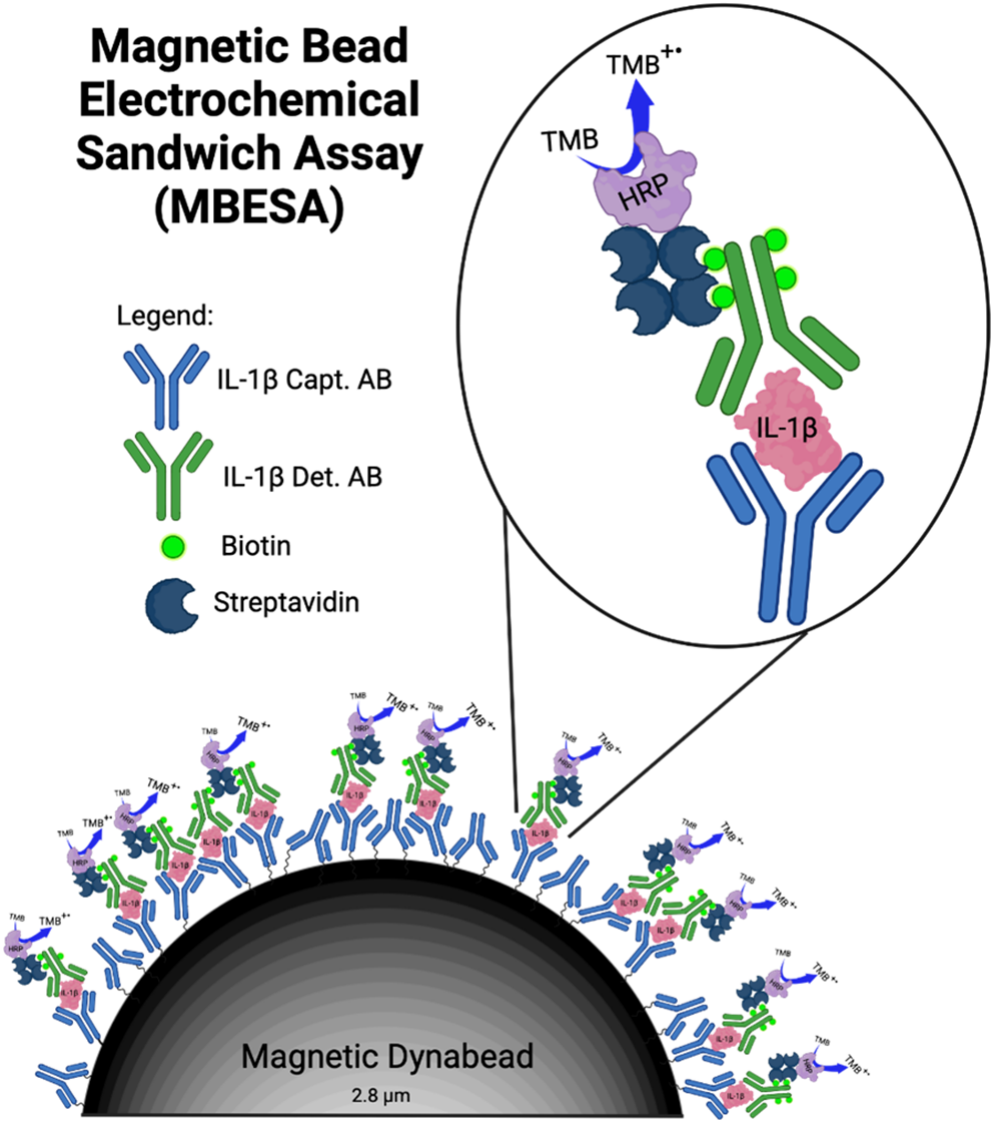
Magnetic Bead Electrochemical Sandwich Assay (MBESA) design for interleukin-1β. This highlights the bioconjugation of IL-1β capture antibodies, IL-1β, IL-1β detection antibodies, and HRP onto magnetic Dynabeads. Image created in BioRender.

It is crucial to characterize and quantify the magnetic Dynabeads, IL-1β capture antibody binding, antibody orientation, and analytical signal from magnetic bead-based immunoassays to advance electrochemical biosensor development and performance. With the use of electron microscopy techniques and an indirect MBESA, we can characterize the surface of the magnetic Dynabeads and indirectly quantify the immunoassay bioconjugation which yields electrochemical signal. Herein, we present a comprehensive electron microscopy investigation of the magnetic Dynabead immunoassay to provide quantifiable data on the formation of the immunoassay, offering novel insights into optimizing bead binding and enhancing electrochemical immunoassay efficiency. The novelty of this work lies in its rigorous and detailed electron microscopy characterization, which serves to bridge the gap between immunoassay design and the physical understanding of their nanoscale architecture.

## Methods

### Materials

Human Interleukin-1β Matched Antibody Pair kit (#CHC1213), Magnetic Dynabeads Antibody Coupling Kit (#14311D), Goat anti-Human IgG Fc Secondary Antibody, Biotin (#13–4998–83), poly-HRP Streptavidin (#N200), DynaMag-2 magnet stand (#12321D) and a HulaMixer (#15920D) were purchased from Thermo Fisher Scientific. 3,3′,5,5′-tetramethylbenzidine (TMB) Liquid Substrate System (Cat. #T0440) was purchased from Sigma-Aldrich and BioReady Gold Nanospheres-Strep-40 nm (#AUIR40, 10 OD) were purchased from NanoComposix, LLC. Ethical compliance: no human subjects or animal studies were involved in this work. All biological materials, including antibodies and cytokines, were commercially obtained from certified vendors and were not derived from identifiable human or animal samples; therefore, Institutional Review Board (IRB) or Institutional Animal Care and Use Committee (IACUC) approval was not required. ***Caution!*** Safety Considerations: Biological agents such as antibodies and cytokines were handled following BSL-2 protocols.

### Instrumentation

Electrochemical measurements were performed with a CHI 1440 4-Channel Potentiometer/Potentiostat (CH Instruments, Inc., Austin, TX) with amperometry parameters: −0.2 V potential, 0.5 s sample interval, 20 s sampling time, 0 s quiet time, and 1 × 10^–5^ A/V sensitivity. Carbon screen-printed electrodes were obtained from Pine Research Instrumentation, Inc. (#RRPE1001C; Durham, NC) and had a 2 mm diameter carbon working electrode, a Ag/AgCl reference electrode, and a carbon counter electrode. Sensor performance was evaluated via amperometric *i*-t curves. These curves were integrated using CHI software, giving charge, and the total charge was taken as the sensor output value.

Scanning electron microscopy (SEM) was performed on a Zeiss Merlin System operating with a probe current of 100 pA, an accelerating voltage of 2 kV, and a working distance of 5 mm. Secondary electron (SE) imaging was conducted using both an SE1 and Everhart-Thornley SE2 detector. The SE1 detector collected electrons emitted directly from the sample surface, providing higher resolution topographical detail, whereas the SE2 detector collected both surface and scattered secondary electrons, yielding increased signal intensity and overall image brightness. Further imaging was performed using a Tecnai G2 Osiris system for scanning/transmission electron microscopy (S/TEM). Image analysis, including quantification of energy-dispersive X-ray (EDX) spectra and processing of STEM-EDX maps, was conducted using Bruker Esprit software.

### Magnetic Bead Electrochemical Sandwich Assay for Electron Microscopy

This MBESA, an adaptation of our laboratory’s previous studies that have validated the immunoassay design, is divided into separate parts for electron microscopy characterization.^12^ To investigate bioconjugation, each step of the MBESA was conducted simultaneously in individual sample holders. Human IL-1β capture antibody (20 μg) was allowed to bind to 1 mg of Magnetic Dynabeads for 24 h at 37 °C. Beads were then resuspended in 100 μL of SB Magnetic Dynabeads Kit solution. Lastly, stock bead solution (10 μL) was aliquoted into individual microcentrifuge tubes.

Following capture antibody immobilization and bead preparation, lyophilized human IL-1β was resuspended and diluted to 500 pg/mL in PBS. A concentration of 500 pg/mL was selected to reflect physiologically relevant inflammatory levels, while also enabling optimal electron microscopy characterization. Human IL-1β (20 μL) and 250 ng/mL of human IL-1β detection antibody (2 μL) were added to three bead-aliquoted microcentrifuge tubes and allowed to bind for 30 min. Poly-HRP Streptavidin (15 μL of 4 μg/mL) was then added to two bead-aliquoted microcentrifuge tubes and allowed to bind for 5 min. Lastly, TMB (100 μL) was added to one of the bead-aliquoted microcentrifuge tubes and allowed to react for 2.5 min. All binding reactions occurred at room temperature on a HulaMixer with a series of PBS rinses between each binding addition. This yielded unique samples: magnetic Dynabeads + IL-1β capture antibody (Sample 1), magnetic Dynabeads + IL-1β capture antibody + human IL-1β + IL-1β detection antibody (Sample 2), magnetic Dynabeads + IL-1β capture antibody + human IL-1β + IL-1β detection antibody + poly-HRP (Sample 3), and magnetic Dynabeads + IL-1β capture antibody + human IL-1β + IL-1β detection antibody + poly-HRP + TMB (Sample 4). To enhance microscopy analysis, 500 μL of 40 nm streptavidin-conjugated gold nanoparticles (BioReady Gold Nanospheres) were added to bind to the IL-1β detection antibody. This conjugation consisted of magnetic Dynabeads + IL-1β capture antibody + human IL-1β + IL-1β detection antibody + gold nanoparticles (AuNP) (Sample 5).

### Direct Magnetic Bead Electrochemical Sandwich Assay

For comparison of magnetic bead bioconjugation and electrochemical response, human IL-1β capture antibody at 3 μg, 5 μg, and 10 μg were each allowed to bind to 1 mg Magnetic Dynabeads for 24 h at 37 °C. Each bead set was resuspended in 100 μL of SB Magnetic Dynabeads Kit solution, and 10 μL of the stock bead suspension was aliquoted in triplicate into microcentrifuge tubes. Human IL-1β (100 μL) diluted to 1000 pg/mL in PBS and 1000 ng/mL of human IL-1β biotinylated detection antibody (5 μL) were then added to each tube and incubated for 30 min. Poly-HRP Streptavidin (15 μL of 4 μg/mL) was subsequently introduced and incubated for 5 min, followed by the addition of TMB (100 μL), which reacted for 2.5 min prior to off-bead electrochemical analysis. All binding reactions occurred at room temperature on a HulaMixer with a series of PBS rinses between each binding addition. Electrochemical measurements were subsequently taken of each microcentrifuge tube for all concentrations.

### Indirect Magnetic Bead Electrochemical Sandwich Assay

For characterization and relative quantification of the capture antibody orientation on the magnetic beads, the MBESA design was fabricated into an indirect format. Human IL-1β capture antibody at 3 μg, 5 μg, and 10 μg, were each allowed to bind to 1 mg of Magnetic Dynabeads for 24 h at 37 °C. Each set of beads was resuspended in 100 μL of SB Magnetic Dynabeads Kit solution. For each capture antibody loading (3 μg/mg beads, 5 μg/mg beads, and 10 μg/mg beads), 10 μL of the stock bead solution was aliquoted in triplicate to microcentrifuge tubes. For each microcentrifuge tube of 10 μL bead solution, 10 μL of 500 ng/mL Goat anti-Human IgG Fc Biotinylated Secondary Antibody was added and allowed to bind for 30 min. Next, 15 μL of 4 μg/mL poly-HRP streptavidin was added and incubated for 5 min, followed by the addition of 100 μL TMB, which was allowed to react for 2.5 min. All binding reactions occurred at room temperature on a HulaMixer with a series of PBS rinses between each binding addition. Electrochemical measurements were subsequently taken of each microcentrifuge tube for all concentrations.

### Scanning Electron Microscopy

To assess morphology and surface features of the immunoassay bead foundation, high-resolution SEM was employed. Magnetic Dynabeads (0.5 mg) were diluted in 1 mL deionized water and prepared for SEM. MBESA Samples 1–5 were each rinsed three times with deionized water, resuspended in 1 mL deionized water, and prepared for imaging. The resulting suspensions were cast onto carbon tape and imaged using a Zeiss Merlin system.

### Scanning Transmission Electron Microscopy–Energy Dispersive X-ray Spectroscopy

To enhance visualization of the spatial distribution and loading of the immunoassay on the magnetic beads, streptavidin-conjugated AuNPs were added to the MBESA. These nanoparticles bind specifically to the biotinylated detection antibody, which is attached to the target protein and the capture antibody coated beads. Magnetic Dynabeads (0.5 mg) were diluted in 1 mL deionized water and prepared for STEM-EDX. Replicates of MBESA Sample 2 were rinsed 3 times in deionized water, resuspended in 1 mL deionized water, and prepared for STEM-EDX. Replicates of MBESA Sample 5 with AuNPs were rinsed 3 times in deionized water, resuspended in 1 mL deionized water, and prepared for STEM-EDX. All STEM-EDX samples were transferred to carbon type-B, 400 mesh, copper grids with an approximate hole size of 42 μm (Ted Pella, # 01814-F) by briefly dipping for three rounds of three second immersions and imaged on a FEI Tecnai G2 Osiris system.

## Results and Discussion

Capture antibody immobilization on magnetic Dynabeads can occur in random orientations and is often influenced by empirical factors rather than precise calculations. Random orientation and high steric hindrance can lead to unintended consequences, such as denaturation of the antibody, blocking of the antigen-binding site, or weak attachment that results in loss during washing.^20–22^ These factors can reduce immunoassay sensitivity and the readout signal by decreasing the bioactivity of immobilized antibodies.^4,23^ Therefore, thorough characterization of antibody orientation and loading is critical to maximize immunosensor performance.

The current designed MBESA is highly sensitive, specific, and reproducible.^16^ However, the current assembly does not enforce site-specific binding, which can result in random orientation of the fragment antigen-binding (Fab) region located on the two arms of the Y-shaped antibody molecule. Thus, characterizing the relative antibody orientation with an indirect MBESA can inform the future development of sensors that preferentially bind Dynabeads via the fragment crystallizable (Fc) region, the tail-like portion of the antibody, to fully optimize electrochemical performance and analyte sensitivity.^24,25^

To investigate the orientations of the Fab binding sites in this system, we employed an indirect MBESA, which is conceptually similar to an indirect ELISA. In this assay, the capture antibody was immobilized on the Dynabead surface, and binding of this antibody was detected using a secondary antibody conjugated to electrochemical reporters, HRP and TMB. This design allowed quantification of antibody attachment while providing insight into random antibody orientation on the surface, because the secondary antibody can only bind to the primary antibody when the Fc region is exposed. Consequently, the indirect MBESA reveals the relative fraction of capture antibodies oriented with their Fab regions “down”, directly linking antibody orientation to electrochemical sensor performance (Figure 2). To complement the electrochemical evidence of variable Fc accessibility, and therefore antibody orientation of the indirect MBESA (Figure 3), we applied a direct MBESA, a direct AuNP MBESA, and high-resolution SEM and STEM-EDX imaging (Figures 4–7). These combined approaches allowed enhanced visualization and elemental confirmation of successful immunoassay bioconjugation, while providing insight into functional orientation variability.

**Figure 2:**
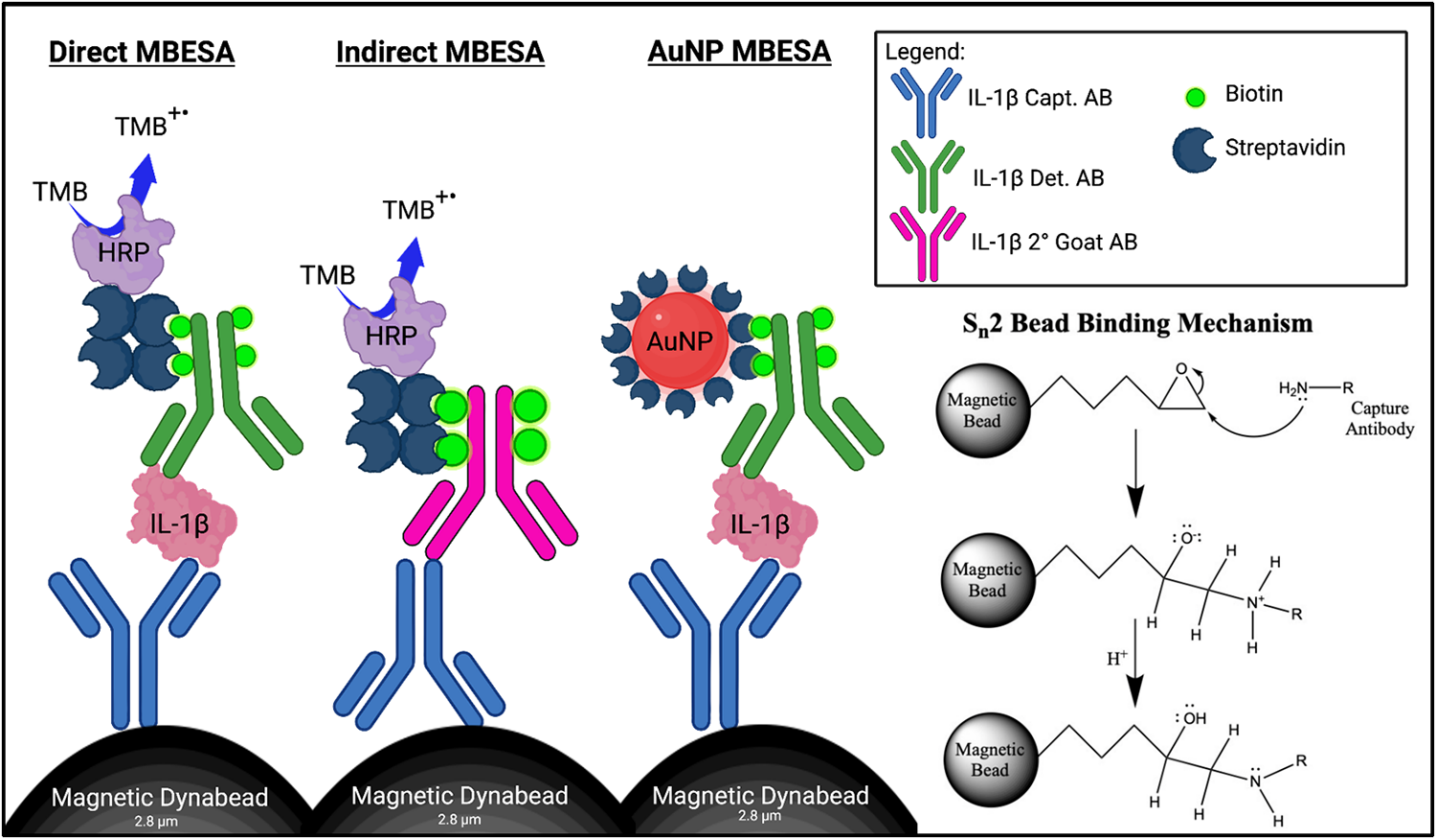
Magnetic Bead Electrochemical Sandwich Assay (MBESA) adapted with a gold nanoparticle for microscopy analysis and to an indirect assay with a secondary antibody for relative antibody orientation confirmation. Capture antibody binds to magnetic Dynabead via an S_N_2 reaction mechanism. Image created in BioRender.

**Figure 3:**
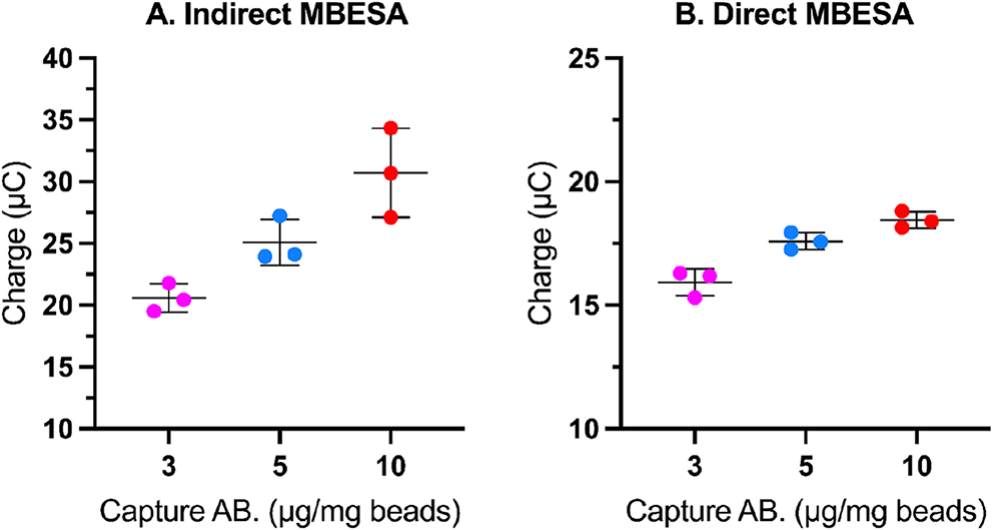
Indirect and direct Magnetic Bead Electrochemical Sandwich Assays (MBESAs) were examined in triplicate, highlighting sample means and standard deviations. All indirect MBESAs (panel A) were performed with 10 μL of 500 ng/mL Goat anti-Human IgG Fc Secondary Antibody (5 μg/assay), while direct MBESAs (panel B) were performed with 5 μL of 1000 ng/mL of Human IL-1β Detection Antibody (5 μg/assay). The direct MBESA signal corresponds to the detection antibody binding to the IL-1β antigen, while the indirect MBESA signal corresponds to the secondary antibody binding to the Fc region of the capture antibody.

**Figure 4:**
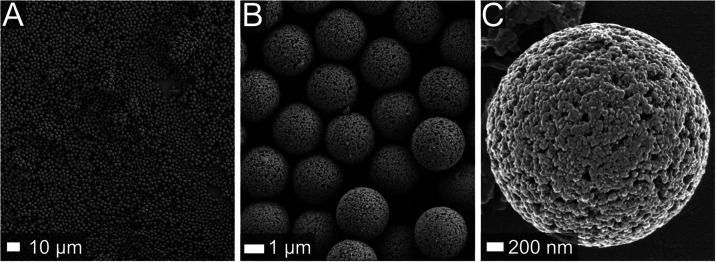
Scanning electron micrographs of bare Dynabeads demonstrating their monodispersity (image A) and their surface topography (images B and C). Images A and B were acquired using the SE2 detector, a contrast mode sensitive to material composition, resulting in darker iron oxide cores, whereas image C was acquired using the SE1 detector, a surface sensitive mode that highlights fine topographical details and bead edges.

It is important to note that for electrochemical studies, capture antibody loadings of 3–10 μg/mg beads were selected to maximize functional signal output while maintaining reproducibility. In contrast, higher capture antibody loading (20 μg/mg beads) was employed for electron microscopy experiments to promote surface coverage and enhance structural visualization of immunoassay assembly. Likewise, antigen and detection antibody concentrations used in electrochemical measurements were selected to achieve amperometric signal maximizing conditions, whereas microscopy studies were performed under conditions to enhance bioconjugation visualization.

### Indirect and Direct Magnetic Bead Electrochemical Sandwich Assay

To quantify the exposed Fc regions on antibodies immobilized on magnetic Dynabeads, an indirect MBESA was utilized. This approach allowed evaluation of the relative proportion of capture antibodies that are oriented in the Fab-up position and the Fab-down position, as shown in Figure 3A. When capture antibodies are bound in a Fab-down orientation, the antigen-binding sites are partially or fully blocked, preventing the IL-1β from binding and thereby reducing electrochemical signal and overall sensor performance.^24^ This analysis of the indirect MBESA shows how antibody orientation affects sensor efficiency and provides quantitative insight into the fraction of antibodies available for effective antigen capture.

The indirect MBESA results revealed high variability among replicates, as indicated by the increased standard deviations, suggesting inconsistent Fab-down orientations of the capture antibodies. This proof-of-principle experiment underscores the need to develop site-specific electrochemical sensors that preferentially immobilize antibodies in the Fab-up orientation, potentially using techniques such as supramolecular chemistry to preorganize the surface.^26^ Under the assumption that all IL-1β capture antibodies (molecular weight 150 kDa) are accessible for binding, the electrochemical charge yield is normalized to a maximum value of 40 μC, defined as the highest achievable charge measured experimentally using TMB and HRP under optimized assay conditions, and treated as 100% signal output. However, for 3 μg of capture antibody in triplicate, the average measured signal was 20.6 ± 1.1 μC, corresponding to an average of 52% of antibodies in the Fab-down orientation. Similar trends were observed for 5 μg (average charge = 25.1 ± 1.9 μC; average of 63% Fab-down) and 10 μg (average charge = 30.7 ± 3.6 μC; average of 77% Fab-down), with these percentages reflecting relative binding accessibility rather than absolute fractions. Collectively, these findings confirm that conventional immobilization strategies on Dynabeads rarely produce uniform Fab-up orientations and underscore the need for site-specific approaches to maximize exposed antigen-binding sites.

In contrast to the indirect MBESA, the direct MBESA shown in Figure 3B was designed as a sandwich assay in which a biotinylated detection antibody bound directly to target IL-1β, providing robust antigen recognition and highly reproducible signal output. The direct and indirect MBESAs were prepared from identical magnetic bead batches to enable consistent experimental comparisons between assay formats. Using capture antibody concentrations of 3 μg/mg beads, 5 μg/mg beads, and 10 μg/mg beads (*n* = 3 each), the produced direct MBESA average charge outputs were 15.9 ± 0.5 μC, 17.6 ± 0.4 μC, and 18.4 ± 0.3 μC, respectively. The comparatively small standard deviations of the direct MBESA design represent up to an order of magnitude improvement in signal reproducibility relative to the indirect format.^16^ This low variability across replicates is attributable to the consistent availability of antigen-binding sites in the sandwich format; however, because of the detection antibody’s binding mechanism, the direct MBESA alone does not provide information on the orientation of immobilized capture antibodies. Together, the indirect and direct MBESA approaches complement one another: the indirect format provides quantitative insight into heterogeneous Fab-down orientation, while the direct format delivers highly reproducible, sensitive detection of IL-1β binding. Integration of these methods, particularly when combined with high-resolution EM and AuNP labeling, allows both functional and structural characterization of the bioconjugated Dynabeads, guiding the rational design of next generation immunosensors.

Ultimately, the electrochemical signal reflects functional binding availability rather than rigid molecular positioning. Antibodies exhibit hinge flexibility, dynamic conformations, and steric hindrances, that can all influence apparent Fab or Fc accessibility independent of true orientation. Thus, this analysis serves as an experimental framework for comparative assay optimization, consistent with prior studies emphasizing that antibody orientation metrics in immunoassays represent functional trends rather than fixed structural angles.^20–22^ Recognizing these functional and structural limitations highlights the importance of developing and evaluating immobilization strategies designed to more deliberately control antibody orientation.

There have been strategies to alter antibody orientation to increase the availability of Fab-up regions and exposed binding sites; however, many of these techniques rely on flat substrates and methods such as protein A mediated immobilization or microarray style surface modification chemistries.^27,28^ Similarly, while methods such as size-exclusion chromatography-fluorescent detection (SEC-FLD)^29^ have provided valuable characterization of protein conjugation on magnetic Dynabeads, they typically rely on advanced instrumentation and highly trained personnel, complicating routine implementation. Beyond these methodological constraints, the inherent complexity of immunoassay bioconjugation introduces further variables that affect sensor performance. In the MBESA system, for example, there may be three to five biotin molecules (molecular weight: 244 g/mol) per detection antibody, three streptavidin molecules (molecular weight: 55 kDa) per HRP enzyme, and up to four biotin binding sites per single streptavidin molecule (Figure 2). Each added bioconjugate increases steric hindrance and can obstruct nearby binding sites, further contributing to inconsistent antibody orientation and reduced antigen accessibility. Collectively, these insights emphasize the need for strategies that intentionally orient antibodies in a site-specific, Fab-up configuration to optimize electrochemical sensor performance. The indirect MBESA provides a straightforward, yet powerful foundation for guiding the development of such next generation bioconjugation approaches.

### Scanning Electron Microscopy

For immunoassay sensor applications, it is important to evaluate antibody binding interactions and potential steric hindrance to ensure efficient target capture and strong analytical response. Scanning electron microscopy (SEM) provides a detailed approach to examine the surface of Dynabeads and assess surface features associated with bioconjugation. As shown in Figure 4, scanning electron micrographs confirm the monodispersity and general uniformity of the bead substrates. The images collected with the Everhart-Thornley SE2 detector (Figure 4A, 4B) emphasize compositional contrast, allowing the iron oxide cores to appear darker and more distinguishable from the surrounding polymer matrix. In contrast, the SE1 detector image (Figure 4C) highlights subtle topographical features at the bead surface, offering enhanced edge definition that supports interpretation of surface texture and potential biomolecule coverage. Together, these complementary imaging modes provide a more complete visualization of the surface features of the MBESA foundation and support SEM as a foundational tool in the bottom-up characterization of the MBESA platform.

### Scanning Transmission Electron Microscopy-Energy Dispersive X-ray Spectroscopy

To directly visualize and quantitatively assess the surface chemistry associated with immunoassay assembly, STEM-EDX was employed. In the MBESA platform, primary IL-1β capture antibody was covalently immobilized on the magnetic Dynabead, followed by binding of the target IL-1β and subsequent recognition by a biotinylated-detection antibody, forming a classic sandwich structure. A streptavidin-coated gold nanosphere was then introduced, attaching through the very strong, noncovalent biotin–streptavidin interactions.^30^ Representative spectra and elemental maps are shown from three isolated Dynabeads per condition; however, all spectra were reproducible across multiple beads within 0.5 mg samples prepared in independent experiments, confirming bioconjugation.

It is important to consider the chemical composition of the original Dynabead substrate, and the chemical differences present on the substrate after successful bioconjugation. Figure 5A, 5B showcase an approximate 3-fold increase in sulfur density across three isolated Dynabeads, which was quantified using the EDX spectrum in Figure 6 normalized to iron content. For clarity, the low-level sulfur signal observed on bare Dynabeads (0.2 atomic percent) is attributed to trace residual sulfur associated with the commercial bead manufacturing process and inherent polymer surface chemistry. The Dynabeads used in this study are epoxy coated beads with a polystyrene shell produced via a proprietary process, and such polystyrene based magnetic beads can contain minor sulfur contributions from polymerization initiators, stabilizers, or other trace additives introduced during large scale synthesis and coating.^15^

**Figure 5:**
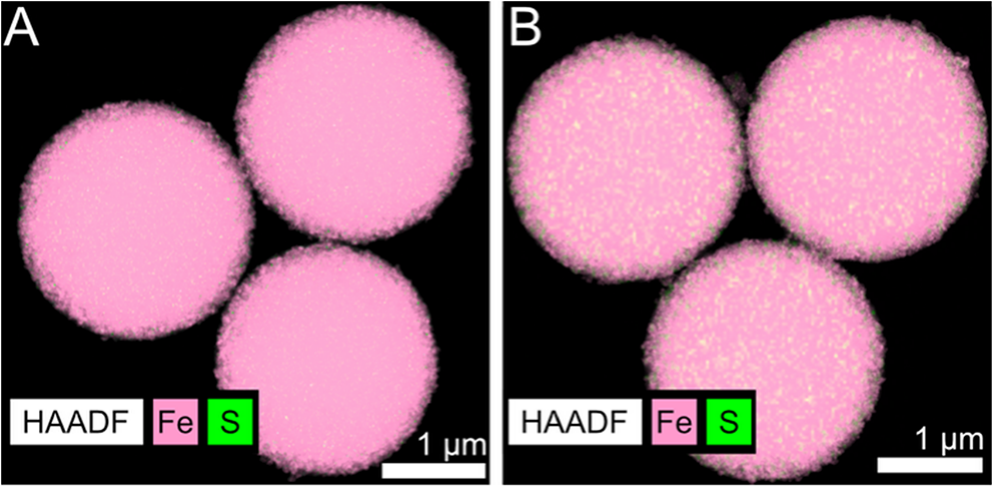
STEM-EDX of Dynabeads. Images A and B showcase the increase in sulfur content before and after the MBESA assay, respectively, from three isolated Dynabeads per condition.

**Figure 6:**
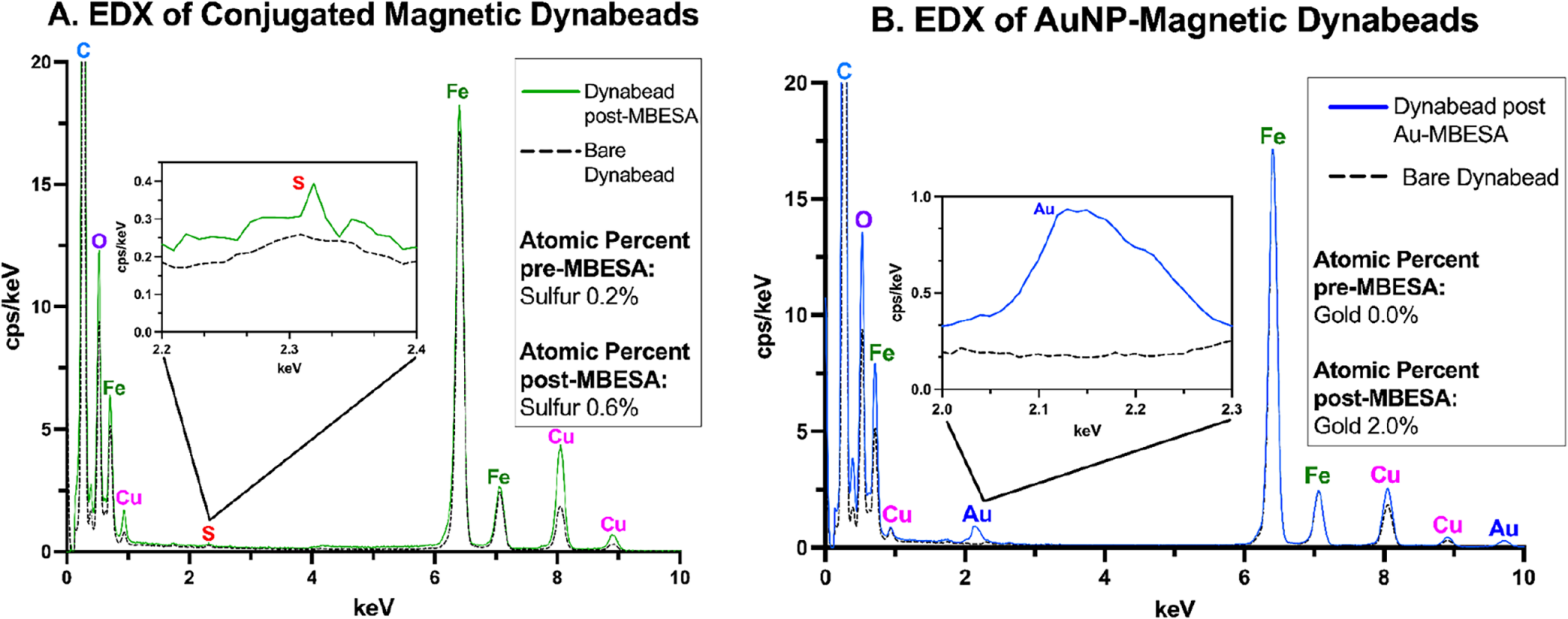
Energy-dispersive X-ray spectroscopy (EDX) of three isolated Dynabeads highlighting elements relevant to the MBESA assay. Panel A shows the sulfur signal at 2.3 keV, used to track the addition of sulfur-containing biomolecules, while panel B displays a pronounced gold peak corresponding to the conjugation of gold nanoparticles to the Dynabeads. All spectra were normalized to the iron peak at 6.3 keV for comparison. Atomic percentages were determined from net integrated peak areas following background subtraction in Bruker ESPRIT.

In contrast, the postconjugation increase in sulfur content is chemically consistent with the introduction of antibody and antigen biomolecules onto the bead surface. Antibodies, composed of two heavy polypeptide chains and two identical light chains linked by disulfide bonds, were conjugated onto the magnetic Dynabeads, and an increase in sulfur content was therefore expected.^31^ Specifically, active IL-1β consists of 153 amino acids, 12 antiparallel β-strands, and 24 hydrophobic side chains.^32,33^ Based on the amino acid sequence, each active IL-1β molecule contains eight sulfur atoms. Detectable increases in sulfur signals were observed via STEM-EDX specifically in the fully conjugated MBESA beads (reaching 0.6 atomic percent sulfur), providing evidence of successful biomolecule attachment.

In the fully conjugated MBESA, the EDX spectrum (Figure 6A) showed an approximate tripling of sulfur content, reaching a normalized, atomic percent value of 0.6%. It is important to note that because sulfur is a trace element within an iron dominated spectrum, the visual difference in peak intensity appears subtle on a linear scale. The reported atomic percent increase is derived from net integrated peak area following Bremsstrahlung background subtraction and calibration to iron content in Bruker ESPRIT, rather than peak height alone. For low abundance elements such as sulfur, relatively small absolute increases in net intensity can therefore correspond to larger relative changes in calculated atomic percent due to the low baseline signal. Similar sulfur-to-iron ratios were observed across groups of beads, confirming consistency. Since sulfur was present at trace levels prior to conjugation and remains a low abundance element within an iron dominated spectrum, AuNPs were additionally employed to provide an independent, higher contrast elemental marker for further validation of successful bioconjugation.

To complement sulfur based compositional analysis, AuNP labeling was incorporated as an additional strategy to validate successful bioconjugation, shown in Figure 7. While sulfur provided a compositional marker for biomolecular attachment, the incorporation of AuNPs introduced a distinct and higher contrast elemental signature that further enabled surface visualization. The addition of AuNPs enhanced detection due to the strong gold signal, with an increase of 2% gold content observed in Figure 6B, demonstrating the increased sensitivity achieved with the AuNP MBESA. The corresponding STEM images in Figure 7A–D spatially correlate these elemental changes with the bead surface, with the gold signal highlighted in blue. Figure 7A shows the pre-MBESA bead with a relatively uniform surface, while Figure 7B reveals detection of AuNPs associated with MBESA conjugation. Higher magnification views in Figure 7C, 7D further resolve these AuNPs, demonstrating the successful bioconjugation of antibodies. At nanometer resolution, these images reveal the precise localization of AuNPs and provide a more detailed spatial map of AuNP distribution. Additional transmission electron microscopy (TEM) images of fully conjugated Dynabeads are provided in the Supporting Information for further visualization. These findings represent a meaningful step toward comprehensive characterization of immunoassays and highlight the utility of a bottom-up approach in understanding biomolecular interactions on magnetic Dynabeads.

**Figure 7:**
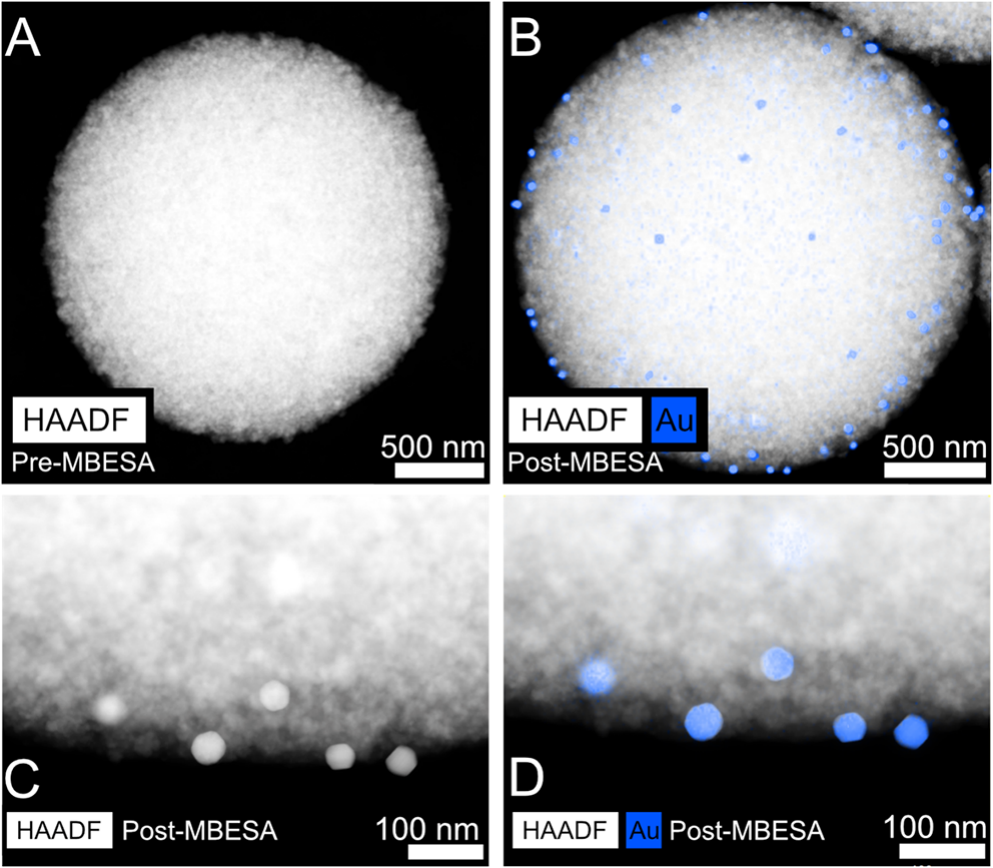
STEM-EDX Gold Analysis of MBESA Dynabeads. Images A and B showcase the increase in gold content before and after the MBESA assay, respectively. Images C and D highlight a high-resolution view of 40 nm gold nanoparticles conjugated to the MBESA.

Collectively, these multimodal analyses demonstrate strong bead-to-bead and sample-to-sample consistency while highlighting the complementary roles of electrochemical and electron microscopy characterization. Imaging of both individual beads and bead clusters revealed consistent sulfur-to-iron ratios, indicating uniform surface modification across the bead population. Notably, the sulfur enrichment became reliably detectable only after completion of the full conjugation sequence, as intermediate stages did not exhibit changes significantly distinguishable from background. Since multiple assay components, including IL-1β, antibodies, and biotins contain sulfur moieties, the observed increase likely reflects cumulative surface accumulation of biomolecular layers rather than sulfur contribution from any single intermediate species. STEM-EDX enabled localized visualization and quantitative elemental comparison before and after conjugation, whereas assay sensitivity and reproducibility were established through direct MBESA electrochemical measurements. In this context, electron microscopy functioned primarily as a structural and compositional validation tool rather than the principal metric of assay efficiency.

Electrochemical analysis of the indirect MBESA quantified the relative proportion of capture antibodies in Fab-down orientation through charge output associated with blocked binding sites, revealing high variability consistent with random surface orientation. In parallel, the AuNP labeled MBESA visualized by SEM and STEM-EDX provided spatial and elemental confirmation of successful detection antibody attachment and the presence of accessible binding regions on the bead surface. Although AuNP imaging does not yield a direct one-to-one correspondence with exact Fab-down percentages, the observed gold distribution patterns support heterogeneous antibody orientation, and therefore the charge variability in the electrochemical findings (Figure 3). Together, these approaches establish a robust analytical framework in which electrochemistry supplies quantitative performance metrics for routine sensor optimization, while electron microscopy offers high-resolution supporting mechanistic and compositional insight into the bioconjugation processes that govern assay behavior.

## Conclusions

In this work, we present a comprehensive characterization of a magnetic bead electrochemical sandwich assay using high-resolution electron microscopy techniques, demonstrating its potential as a powerful platform for electrochemical biosensing applications. By integrating streptavidin-gold nanospheres, we quantified the success of antibody bioconjugation and loading, directly linking nanoscale chemistry to measurable sensor performance. Our analysis revealed that the IL-1β capture antibody binds in heterogeneous orientations, while the full MBESA complex yielded an approximate 3-fold increase in sulfur signal, a key marker of successful conjugation. Beyond confirmation of binding, this study introduces a robust strategy for visualizing bioconjugation on magnetic Dynabeads, advancing the design of more reliable and sensitive immunoassay sensors. Importantly, the complementary use of indirect and direct electrochemical MBESA formats and electron microscopy offers an integrated perspective, connecting functional assay outcomes with structural and compositional insights of surface bioconjugation.

This study contributes to addressing a critical gap in the literature: the pervasive lack of high-resolution electron microscopy investigations into the intricate architecture of magnetic bead-based immunoassays. Our use of electron microscopy, particularly with the incorporation of streptavidin-gold nanospheres, provides unprecedented visual and quantifiable evidence of the bioconjugation process. These gold nanoparticle images serve as direct, high-resolution evidence of antibody and antigen layering on the magnetic beads, offering a tangible understanding of the assay’s physical construction that is often overlooked in traditional immunoassay development. This visual characterization moves beyond mere functional validation, providing crucial insights into the spatial arrangement and density of biomolecules, which directly impacts assay performance and reproducibility.

Our future work includes continually optimizing the MBESA to maximize binding efficiency and enable a multiplex approach for detecting multiple targets, as well as characterizing the binding orientation of targets using a traditional top-down approach. Another potential consideration for future work is the investigation of the long-term stability of bioconjugated layers under various storage conditions and concentrations, as well as the impact of different sample matrices on the structural integrity of the immunoassay components. Overall, this work emphasizes the importance of bottom-up characterization, or examining the molecular and structural details of bioconjugation, alongside top-down assay performance metrics. By combining these perspectives, investigators can uncover otherwise hidden mechanistic factors that critically influence immunoassay behavior, ultimately enabling the development of more robust, sensitive, and reliable diagnostic platforms.

## Supplementary Material



## Notes

Transmission electron microscopy (TEM) characterization of Dynabeads; including bright-field and scanning TEM images showing bead morphology; core–shell architecture, surface roughness; and interfacial matrix between beads (PDF)

## Abbreviations

CCR2: CC chemokine receptor 2
CCL2: CC chemokine ligand 2
CCR5: CC chemokine receptor 5
TLC: thin layer chromatography
SEM: scanning electron microscopy
TEM: transmission electron microscopy
STEM: scanning transmission electron microscopy
SE1: secondary electron 1′s
SE2: secondary electron 2′s
EDX: energy dispersive X-ray spectroscopy; PBS, phosphate buffered saline
IL-6: interleukin-6
IL-1β: interleukin-1β; OoC, organ on-a-chip
PPROM: preterm premature rupture of the membranes
PTB: preterm birth
MBESA: magnetic bead electrochemical sandwich assay
ELISA: enzyme-linked immunosorbent assay
POC: point-of-care
CV: cyclic voltammetry
SWV: square wave voltammetry
EIS: electrochemical impedance spectroscopy
HRP: horseradish-peroxidase
TMB: 3,3′,5,5′-tetramethylbenziidine
AuNP: gold nanoparticle.
