# Supplementary material for: Electron Microscopy Provides Quantitative Insights into Modified Magnetic Bead Layers for Electrochemical Immunoassays
Source: Bioconjug Chem. 2026 Apr 21;37(7):1350–8. doi: 10.1021/acs.bioconjchem.5c00616 (PMC13377598; doi:10.1021/acs.bioconjchem.5c00616)
Supplement: Supplementary file 1 [file bc5c00616_si_001.pdf]

Electron Microscopy Provides Quantitative Insights into Modified Magnetic Bead Layers for Electrochemical Immunoassays

*Hannah A. Richards, William R. Lowery, Andrea K. Locke, David E. Cliffel**

Hannah A. Richards – Department of Chemistry, Vanderbilt University, Tennessee 37235-1822, United States; https://orcid.org/0000-0002-5472-398X

William R. Lowery – Department of Chemistry, Vanderbilt University, Tennessee 37235-1822, United States; https://orcid.org/0000-0002-3063-881X

Andrea K. Locke – Department of Chemistry and Department of Biomedical Engineering, Vanderbilt University, Nashville, Tennessee 37235, United States; https://orcid.org/0000-0002-7357-9688

David E. Cliffel - Department of Chemistry, Vanderbilt University, Tennessee 37235-1822, United States; https://orcid.org/0000-0001-8756-106X; Email: d.cliffel@vanderbilt.edu

SUPPORTING INFORMATION

METHODS

Transmission Electron Microscopy. To complement SEM characterization and provide high-resolution visualization of Dynabeads™ and associated bioconjugates, transmission electron

microscopy (TEM) was performed. Samples were imaged using an FEI Tecnai G2 Osiris system operated at 200 kV accelerating voltage (EHT) with a probe current of 2.56 nA to optimize resolution while minimizing sample damage. Minimum contrast settings were employed to maximize the structural detail captured in the images.

RESULTS AND DISCUSSION

Transmission electron microscopy (TEM) further revealed the internal morphology and fine structural details of the Dynabeads™ and their bioconjugates, complementing the surface topology observed by SEM. **Figure S.1A–C** shows representative TEM images.

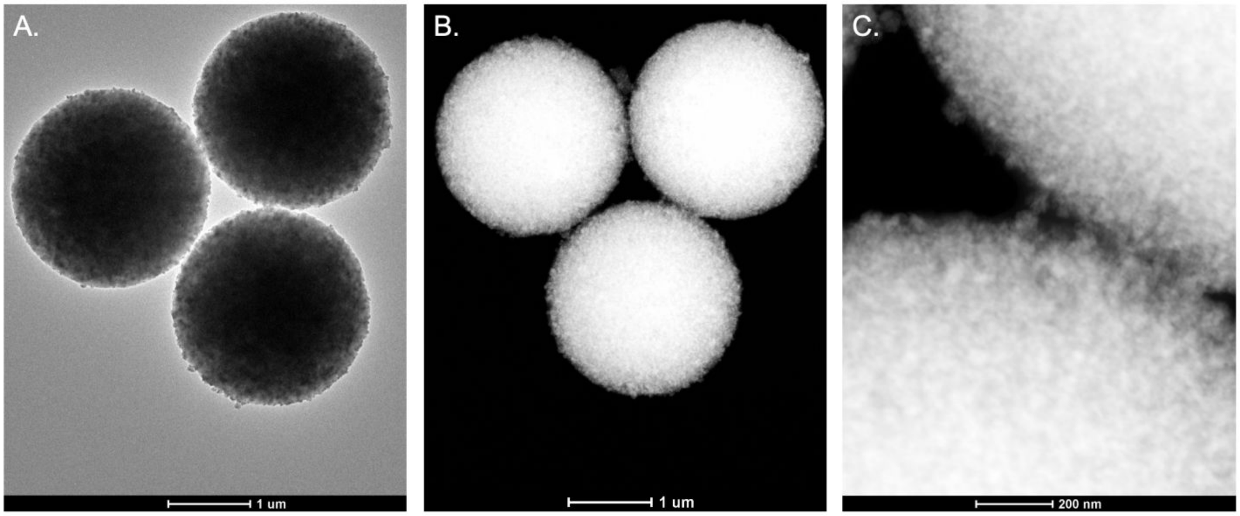

Figure S. 1A-C: TEM characterization of Dynabeads™: (A) bright-field TEM, (B) HAADF-STEM at identical magnification showing contrast inversion of the iron-oxide cores, and (C) high-magnification of the bead surface post-bioconjugation.

Figure S.1A, bright-field TEM, confirms the highly spherical geometry and uniform diameter of the beads, with their dense iron oxide cores appearing dark due to strong electron scattering. **Figure S.1B**, recorded at the same magnification but in scanning TEM mode, inverts the contrast such that the iron-rich cores appear bright white against the polystyrene matrix, highlighting the homogeneous distribution of the thin polymer shell. The highest-magnification image (**Figure**

S.1C) reveals nanoscale surface roughness at the bead interfaces and a thin interfacial matrix linking adjacent beads, potentially arising from surface modification steps. This direct visualization, which is not discernible by SEM alone, supports successful surface functionalization and demonstrates that the core–shell architecture of the Dynabeads™ remains intact following the conjugation protocol.
